# Reliability of CT radiomic features reflecting tumour heterogeneity according to image quality and image processing parameters

**DOI:** 10.1038/s41598-020-60868-9

**Published:** 2020-03-02

**Authors:** Bum Woo Park, Jeong Kon Kim, Changhoe Heo, Kye Jin Park

**Affiliations:** 1 Asan Institute for Life Sciences, University of Ulsan College of Medicine, Seoul, 05505 South Korea; 2 Department of Radiology and Research Institute of Radiology, University of Ulsan College of Medicine, Asan Medical Center, Seoul, Korea

**Keywords:** Predictive markers, Predictive markers

## Abstract

The reliability of radiomics features (RFs) is crucial for quantifying tumour heterogeneity. We assessed the influence of imaging, segmentation, and processing conditions (quantization range, bin number, signal-to-noise ratio [SNR], and unintended outliers) on RF measurement. Low SNR and unintended outliers increased the standard deviation and mean values of histograms to calculate the first-order RFs. Variations in imaging processing conditions significantly altered the shape of the probability distribution (centre of distribution, extent of dispersion, and segmentation of probability clusters) in second-order RF matrices (i.e. grey-level co-occurrence and grey-level run length), thereby eventually causing fluctuations in RF estimation. Inconsistent imaging and processing conditions decreased the number of reliably measured RFs in terms of individual RF values (intraclass correlation coefficient ≥0.75) and inter-lesion RF ratios (coefficient of variation <15%). No RF could be reliably estimated under inconsistent SNR and inclusion of outlier conditions. By contrast, with high SNR and no outliers, all first-order RFs, 11 (42%) grey-level co-occurrence RFs and five (42%) grey-level run length RFs showed acceptable reliability. Our study suggests that optimization of SNR, exclusion of outliers, and application of relevant quantization range and bin number should be performed to ensure the robustness of radiomics studies assessing tumor heterogeneity.

## Introduction

Radiomic data analysis has emerged as a definitive promising technique in medical imaging studies, shifting the diagnosis platform from traditional visual interpretation to high-throughput extraction of quantitative parameters. Supported by sophisticated hardware and software, a huge dataset consisting of multiple radiomics features (RFs) can be extracted from radiological images for quantitative image analysis. In the past decade, radiomics-based approaches have shown great potential for tissue characterization and improving the diagnostic performance in many disease entities^1^.

Tumour heterogeneity is recognized as an important indicator of tumour growth and metastasis, as it is closely related to diverse genetic mutations, metabolic inhomogeneity, hypoxia, and acidosis^2^. Intratumoural heterogeneity can be spatially assessed using cross-sectional imaging data, and RF-based texture analysis can provide voxel-scale information about the compositional distribution profile within a tumour. In this context, several studies have demonstrated the feasibility of RF methods for predicting treatment response and patient prognosis from computed tomography (CT), magnetic resonance imaging and positron emission tomography images^3–7^.

To apply RFs obtained from different institutions and machines in medical practice, their reliability should be guaranteed. Alterations to histogram and probability matrices (the backbones for calculating the first- and second-order RFs, respectively) eventually leads to unstable RF measurements^8^. Several studies have shown that various conditions affecting image quality significantly influence the reliability of RF measurements. For example, inconsistencies across CT scanners such as, spatial resolution, tube current, noise, and reconstruction algorithm may decrease the reliability of CT-derived RFs^9–14^. The reliability of RFs also depends on the techniques used for lesion segmentation, grey-level discretization and quantization range^8,15–18^. Therefore, failure in controlling such confounding factors may lead to inaccurate and unreliable RF estimation^9,15,19–22^.

From this perspective, this study evaluated the effects of quantization range, bin number, signal-to-noise ratio (SNR), and the inclusion of unintended outliers, and assessed how they interact with each other to affect the reliability of RF measurements. To understand the mechanism of RF fluctuations, we performed the alterations to the histogram and second-order RF matrices, to simulate various RF calculation conditions. Thereafter, using representative CT images with different proportions of tumour heterogeneity, we evaluated the reliability of RF value measurements of RF values and the consistency of their inter-lesion ratios using reproducibility statistics. On the basis of the study results, we discuss strategies to improve the reliability of RFs in the evaluation of tumor heterogeneity.

## Results

**Figure 1:**
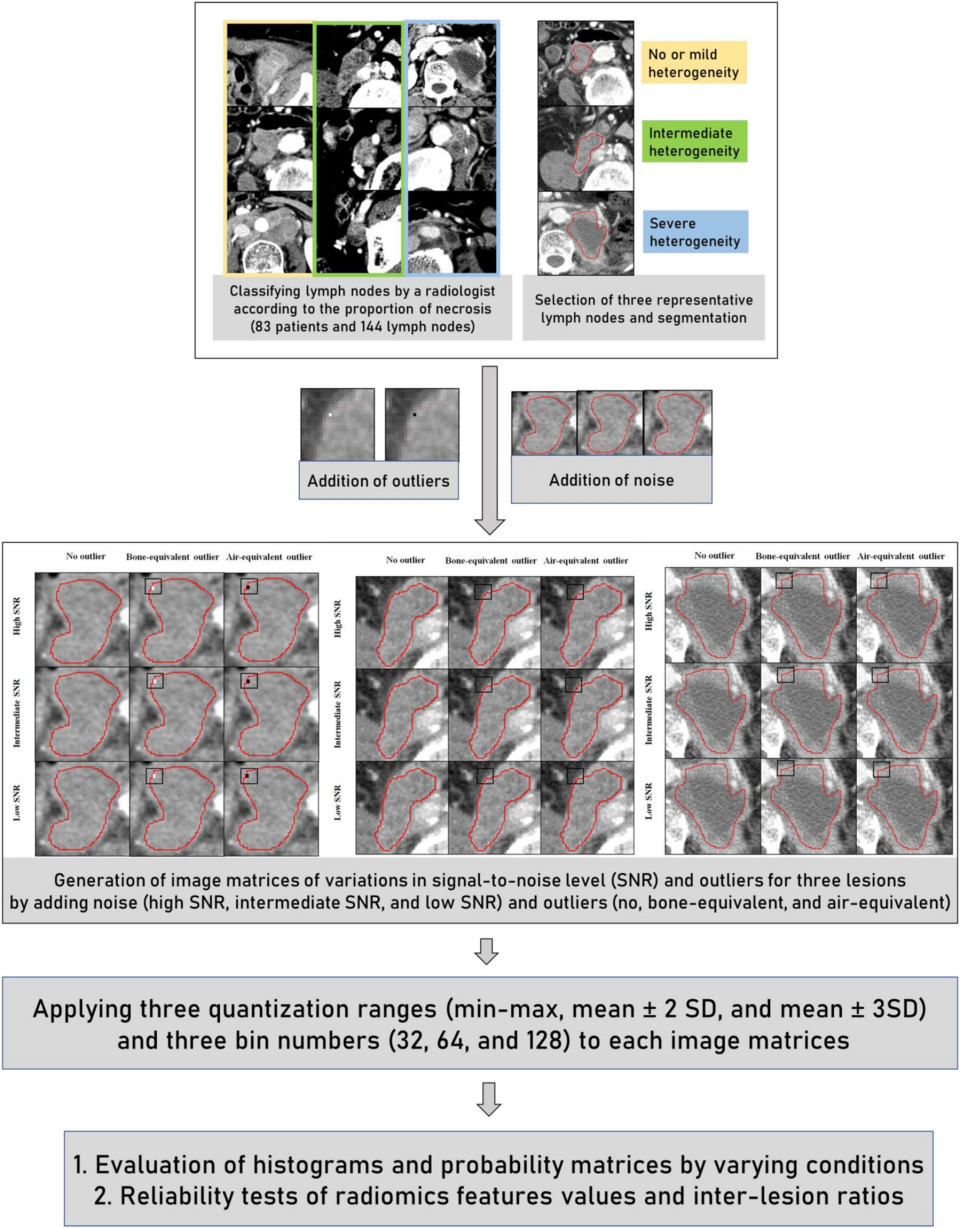
Study processes for lesion selection, image matrix generation, and reliability tests. A radiologist identified three representative patterns of tumour necrosis on the CT images of patients who treated with metastatic urothelial carcinoma. The original CT values in the left upper-boundary of the image matrices were replaced by 2 × 2 voxel clusters of outlying grey value, to evaluate the effect of unintended outlier in the region-of-interest (black squares indicating the location of outliers). By adding noise levels, three image matrices were generated (high, intermediate, and low signal-to-noise level). Consequently, a total of 27 image matrices were generated. These imaging data were quantized by applying various quantization ranges, and bin numbers. Accordingly, reliability for radiomics feature values and inter-lesion ratios were evaluated.

Detailed processes for study processes in regards to lesion selection, image matrices generation, simulation experiments, and reliability tests were summarized in Fig. 1.

### Evaluation of simulation experiments on histograms and probability matrices

#### Histograms

**Figure 2:**
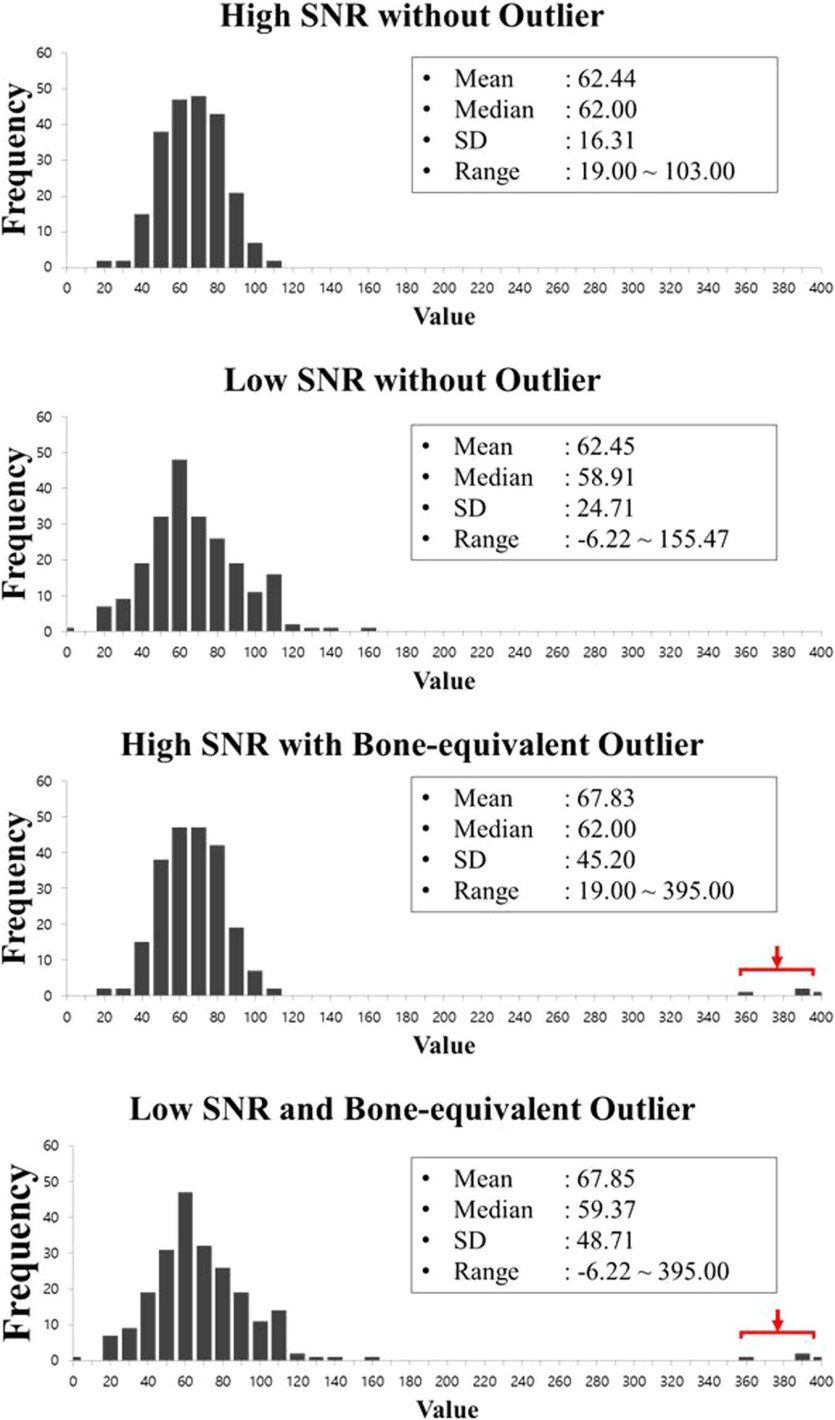
Alterations in the histogram according to SNR and outlier conditions. Histograms from the original CT image (high SNR, (**a**), low-SNR CT image (**b**), outlier-containing high-SNR CT image (**C**), and outlier-containing low-SNR CT image (**d**). Low SNR and unintended outliers (red arrows in (**c**) and (**d**)) increased the SD and range of the histogram. The mean value was increased by the extremely high CT values of bone-equivalent outliers.

The histograms obtained by varying the SNR of the original images and including outlying values are demonstrated in Fig. 2. The addition of noise to the original CT image decreased the SNR and increased the standard deviation (SD) (16.3 Hounsfield unit (HU) for high SNR *versus* 24.7 HU for low SNR) and range (19.0–103.0 HU for high SNR *versus* −6.2–155.4 HU for low SNR). When outliers were added, the mean value and SD increased because of the high CT values of bone-equivalent outliers (mean values of 62.4 HU without outlier *versus* 67.8 HU with outliers; SDs 16.3 HU without outliers *versus* 45.2 HU with outliers). These alterations would affect the measurement of first-order RF values by changing a fraction of the pixels at each grey level (i.e., the essential histogram parameter used for calculating the first-order RFs).

#### GLCM and GLRLM

**Figure 3:**
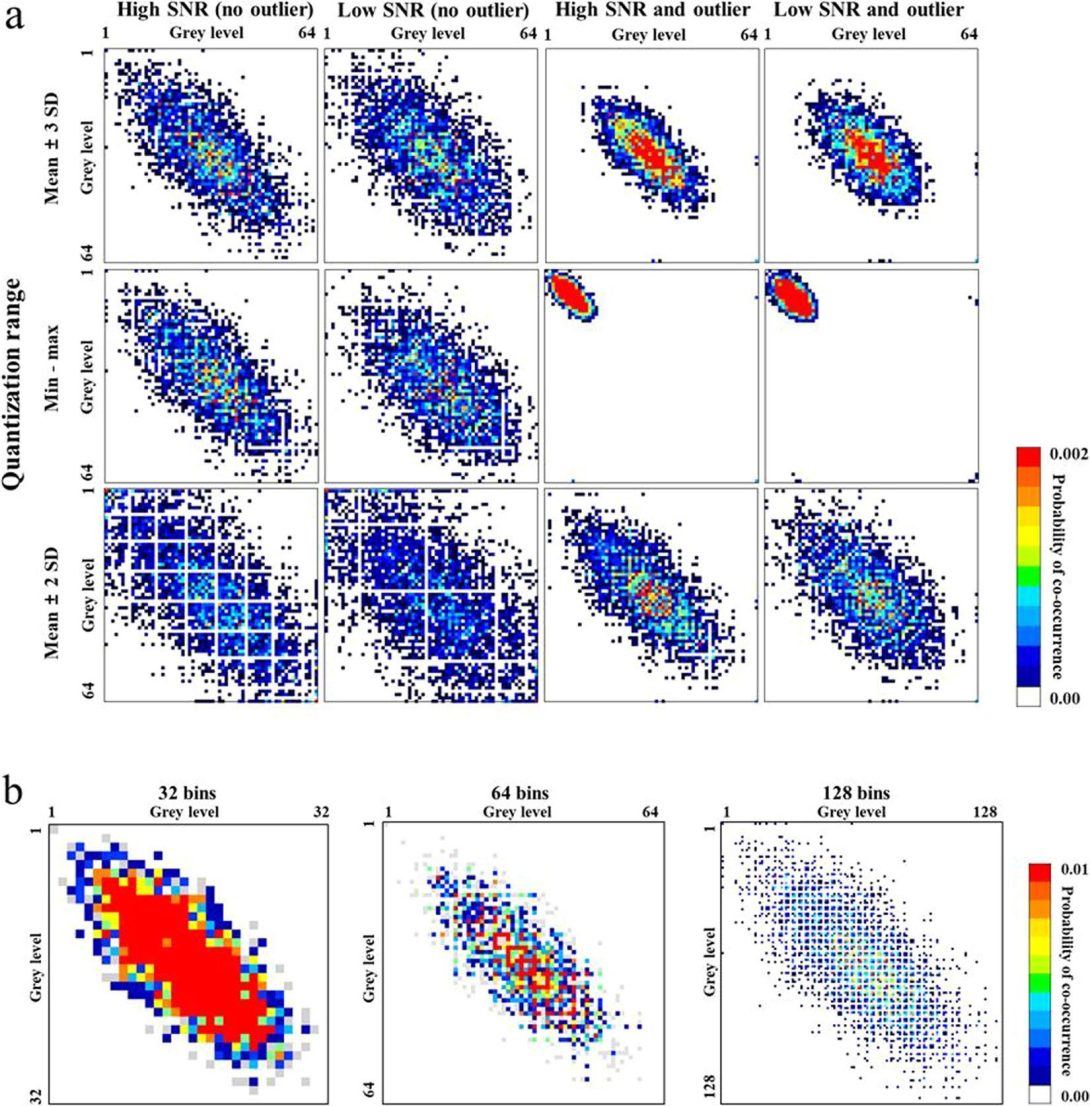
Alterations in the GLCM according to image and image processing conditions. (**a**) The GLCM from the original CT image (high SNR) shows a diagonal probability distribution. The distribution was most concentrated with a distribution of the mean ± 3 SD, and then min‒max, followed by the mean ± 2 SD. Low SNR dispersed the probability distribution. The addition of outliers concentrated the distribution, shifting it towards the upper left direction in the min‒max quantization range. (**b**) Increasing the bin number segmented the probability distribution while maintaining the overall shape.

The probability distribution in the GLCM was altered remarkably by changes in the quantization range, SNR, and outliers, as demonstrated in Fig. 3a. Overall, the probability in the GLCM showed a diagonal distribution, which was more concentrated in the center of matrix than in the periphery. With regards to quantization ranges in high and low SNR conditions, the probability distribution was most concentrated with the quantization range of mean ± 3 SD, followed by min‒max and mean ± 2 SD. When low SNR was applied, the probability distribution was dispersed in all quantization ranges. By contrast, the addition of outliers made the probability distribution markedly concentrated in all quantization ranges. In particular, with the min‒max quantization range, the presence of outliers shifted the centre of the distribution to the upper left direction in the matrix. The use of mean ± 3 SD or mean ± 2 SD did not fully compensate the concentration effect caused by outliers. In the condition of low SNR and inclusion of outliers, the probability dispersion induced by the low SNR was reduced by the concentrating effect of the outliers. The response to bin numbers showed that the greater bin number were chosen, the more segmented the probability distribution became, while the overall distribution shape was maintained (Fig. 3b).

**Figure 4:**
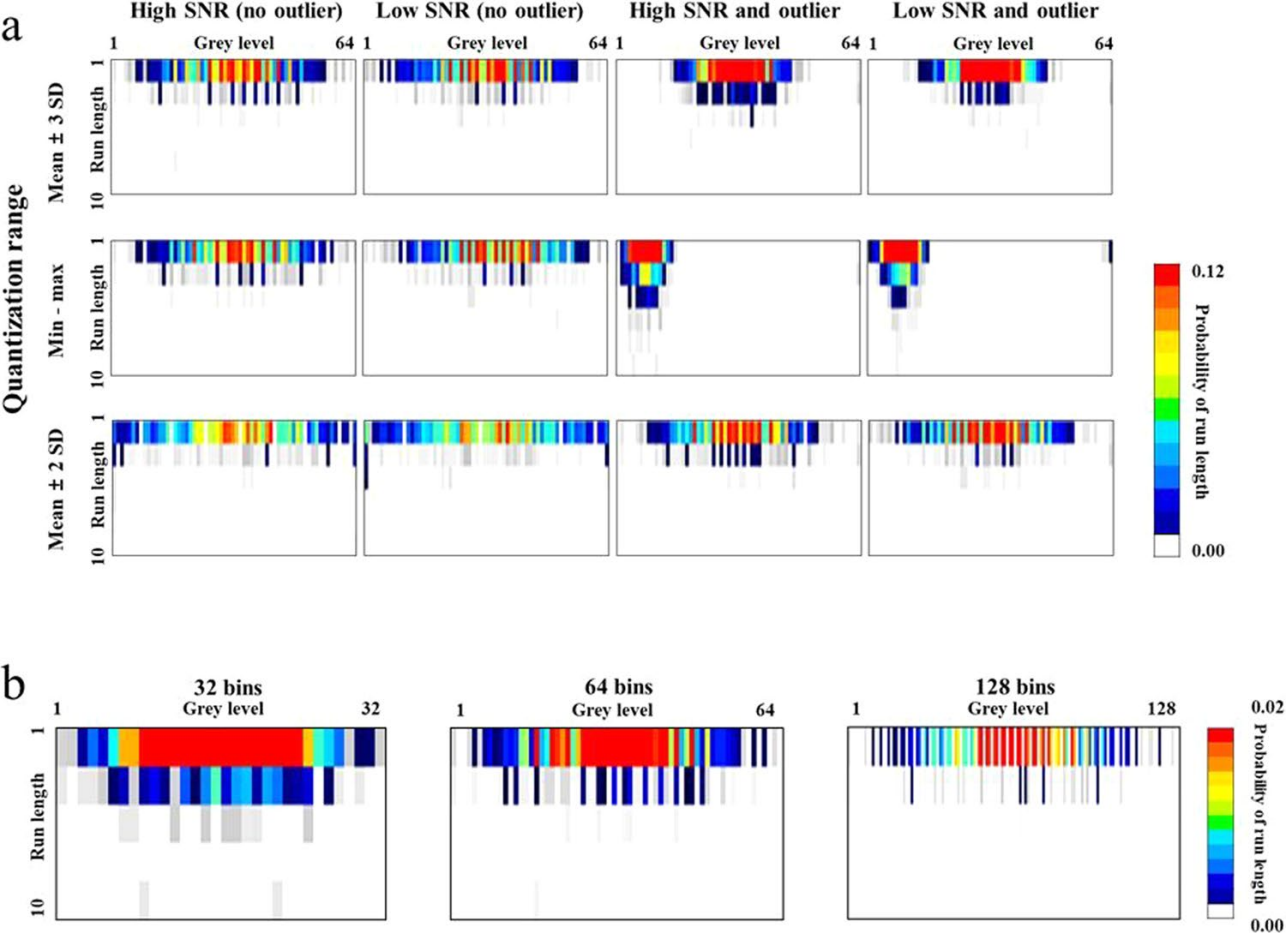
Alterations in the GLRLM according to image and image processing conditions. (**a**) The probability in the GLRLM was distributed mostly in the upper rows as the run length of the original CT image was 3 or less. The distribution was most concentrated with mean ± 3 SD, followed by min-max, and mean ± 2 SD. Low SNR dispersed the probability distribution. The addition of outliers concentrated the distribution and shifted it to the left with the min‒max quantization range. (**b**) Increasing the bin number segmented the probability distribution while maintaining the overall shape.

The probability distribution in the GLRLM was significantly altered by variations in quantization range, SNR, and outliers (Fig. 4a**)**, which changed the GLRLM-derived RF values. The probability values in the GLRLM were distributed mostly in the upper rows because the grey level run length was three or less on the original CT images. Similar to the GLCM, the GLRLM showed the highest probability concentration with the quantization range of mean ± 3 SD, followed by min‒max and mean ± 2 SD. Low SNR dispersed the distribution of the direction of grey levels, while the addition of unintended outliers concentrated the probability distribution in all conditions, shifting it towards the left with the min‒max quantization range. The differences in bin number had an effect similar to that observed in the GLCM, with the distribution being more segmented at higher bin numbers (Fig. 4b).

### Reliability test

#### First-order RFs

In the intra-class correlation coefficient (ICC) assessment, all first-order RFs showed the ICCs ≥ 0.75 for the conditions of quantization range and bin number. By contrast, the conditions of SNR and outliers strongly affected reliability, as ICCs ≥ 0.75 were only shown by five (28%) and three (18%) RFs showing the ICCs ≥ 0.75, respectively (Supplementary Table 1). Under optimal conditions with high SNR and no outliers, all first-order RFs had ICCs ≥ 0.75 (Supplementary Table 2).

In the CV assessment of the consistency of inter-lesion RF ratios (i.e., the ordering of RFs among three lesions representing different proportions of heterogeneity should be consistent under varying conditions), the variation of quantization range option had little effect, as all RFs showed the CVs ≤ 15%. With variable bin number, all parameters except uniformity showed CVs ≤ 15%. However, SNR and outlier options significantly limited the reliability of the inter-lesion RF ratios, with only one (6%) RF (entropy) having a CV ≤ 15% with variable SNR, and all RFs having a CV > 15% with variable outlier options. When these confounding factors were optimized with high SNR and no outliers, the CVs for all parameters were ≤15%, irrespective of the quantization range and bin number options (Supplementary Table 2).

#### GLCM-derived RFs

**Figure 5:**
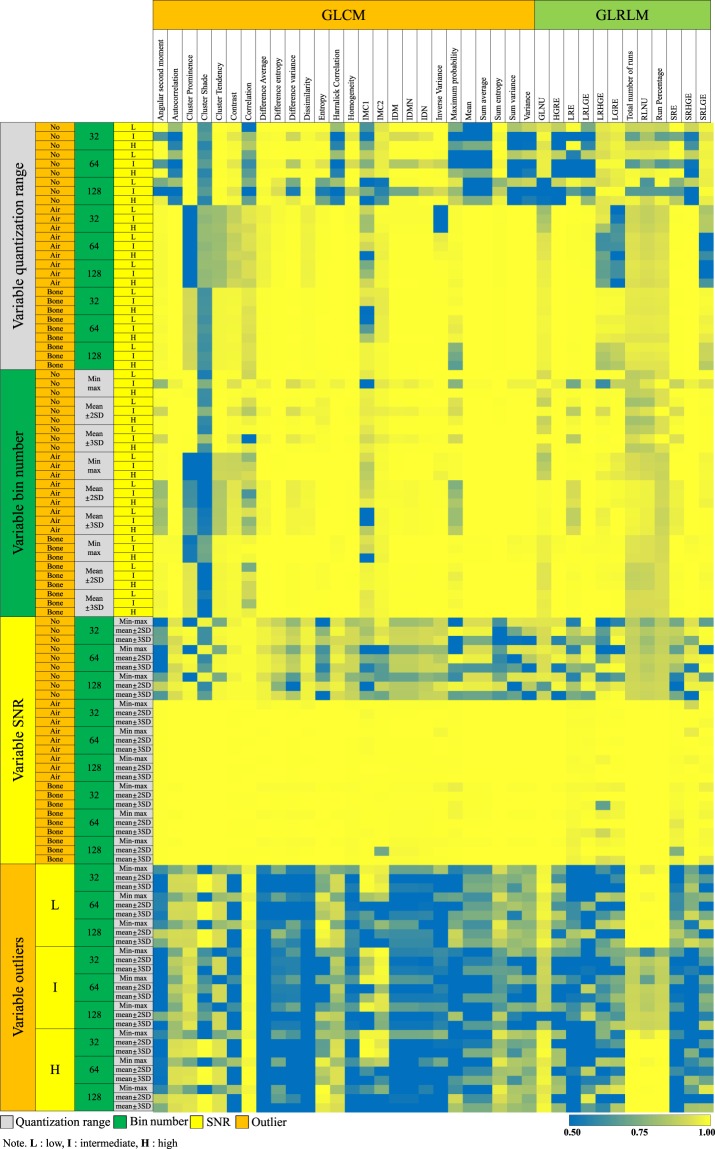
Heatmap showing the ICCs of GLCMs and GLRLMs with image and image processing conditions. The left-most column represents the option being varied, while the second to fourth columns indicates the other options being fixed. The green to yellow colour shows the acceptable range of ICCs (i.e., equal to or greater than 0.75). Abbreviations: IMC = information measure of correlation, IDM = inverse difference moment, IDMN = inverse difference moment normalized, IDN = inverse difference normalized, GLNU = Grey-Level Nonuniformity, HGRE = High Grey-Level Run Emphasis, LRE = Long Run Emphasis, LRLGE = Long Run Low Grey-Level Emphasis, LRHGE = Long Run High Grey-Level Emphasis, LRGE = Low Grey-Level Run Emphasis, RLNU = Run Length Nonuniformity, SRE = Short Run Emphasis, SRHGE = Short Run High Grey-Level Emphasis, SRLGE = Short Run Low Grey-Level Emphasis.

The ICCs for 27 GLCM-derived RFs were influenced by all calculation conditions (Fig. 5). Eight RFs had ICCs ≥ 0.75 (31%) with variable quantization range, 23 (88%) had ICCs ≥ 0.75 with variable bin number, 10 (38%) had ICCs ≥ 0.75 with variable SNR, and one (4%) had an ICC ≥ 0.75 with variable outliers. With high SNR and no outliers, 16 (62%) GLCM-derived RFs showed ICCs ≥ 0.75, regardless of the quantization range and bin number options (Table 1).

**Table 1:** Reliability of GLCM-derived RFs in conditions with high SNR and no outliers.

Radiomic features	ICC		CV (%)	
	Quantization range	Bin number	Quantization range	Bin number
Angular second moment	0.73–0.99	0.99–0.99	0–10	4–41
Autocorrelation	0.80–0.81	0.99–0.99	8–16	0–0
Cluster Prominence	0.99–0.99	0.99–0.99	6–16	0–1
Cluster Shade	0.62–0.64	0.55–0.60	2–87	0–20
Cluster Tendency^*^	0.99–0.99	0.99–0.99	2–7	0–1
Contrast^*^	0.98–0.98	0.98–0.99	3–7	0–1
Correlation	0.50–0.74	0.90–0.98	0–0	0–0
Difference Average^*^	0.99–0.99	0.99–0.99	1–3	0–0
Difference entropy^*^	0.95–0.99	0.99–0.99	0–3	0–6
Difference variance	0.73–0.99	0.99–0.99	2–24	0–27
Dissimilarity^*^	0.99–0.99	0.99–0.99	1–3	0–0
Entropy	0.71–0.99	0.97–0.99	0–2	0–8
Harralick Correlation	0.80–0.81	0.99–0.99	8–16	0–0
Homogeneity^*^	0.99–0.99	0.99–0.99	0–3	0–2
Information measure of correlation^1^	0.42–0.86	0.94–0.95	1–10	6–27
Information measure of correlation^2^	0.52–0.95	0.98–0.98	0–2	1–6
Inverse Difference Moment^*^	0.99–0.99	0.99–0.99	0–5	1–5
Inverse difference moment normalized^*^	0.99–0.99	0.99–0.99	0–5	1–5
Inverse difference normalized^*^	0.99–0.99	0.99–0.99	0–3	0–2
Inverse Variance	0.99–0.99	0.99–0.99	1–41	3–47
Maximum probability	0.46–0.94	0.93–0.99	2–65	8–30
Mean	0.38–0.41	0.99–0.99	4–9	0–0
Sum average	0.38–0.41	0.99–0.99	4–9	0–0
Sum entropy^*^	0.78–0.99	0.99–0.99	0–3	0–4
Sum variance	0.83–0.87	0.99–0.99	8–17	0–0
Variance^*^	0.89–0.90	0.99–0.99	7–15	0–0

Note.—*RFs with ICCs ≥ 0.75 and CVs of ≤15%.

**Figure 6:**
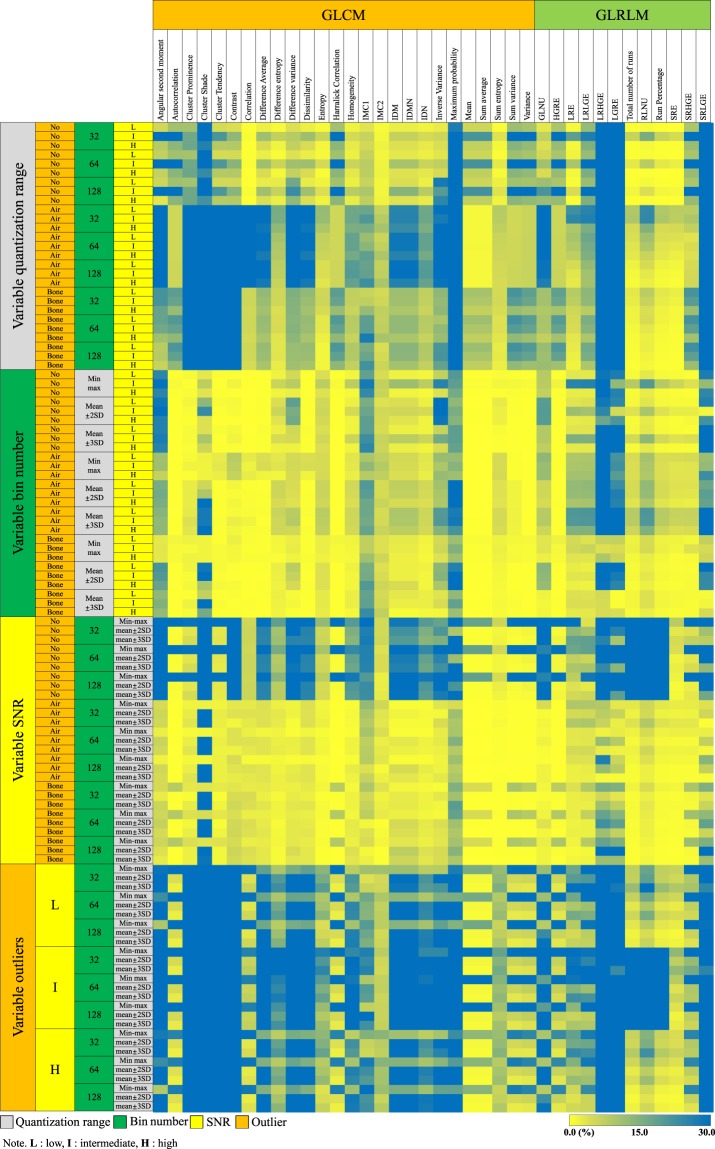
Heatmap presenting the CVs of inter-lesion ratios in the GLCMs and GLRLMs with varying image and image processing conditions. The left-most column represents the option being varied, while the second to fourth columns indicate the other options being fixed. The yellow to green colour shows the acceptable range of CV (i.e. equal to or less than 15%). Abbreviations: IMC = information measure of correlation, IDM = inverse difference moment, IDMN = inverse difference moment normalized, IDN = inverse difference normalized, GLNU = Grey-Level Nonuniformity, HGRE = High Grey-Level Run Emphasis, LRE = Long Run Emphasis, LRLGE = Long Run Low Grey-Level Emphasis, LRHGE = Long Run High Grey-Level Emphasis, LRGE = Low Grey-Level Run Emphasis, RLNU = Run Length Nonuniformity, SRE = Short Run Emphasis, SRHGE = Short Run High Grey-Level Emphasis, SRLGE = Short Run Low Grey-Level Emphasis.

The reliability of inter-lesion RF ratios also strongly depended on the conditions (Fig. 6). Only one RF (4%) showed a CV ≤ 15% with variable quantization range, while 18 (69%) showed CV ≤ 15% with variable bin number, and three (12%) showed CV ≤ 15% with variable SNR. No RF showed a CV ≤ 15% for variations in outliers. In the optimal condition of high SNR and no outliers, 16 (62%) RFs exhibited CVs ≤ 15%, regardless of the quantization range and bin number options.

With inconsistent SNR and outlier conditions, no GLCM-derived RF showed reliable measurement of individual values or inter-lesion ratios (i.e. ICC ≥ 0.75 and CV ≤ 15%). However, under the optimal condition of high SNR and no outliers, 11 (42%) GLCM-derived RFs demonstrated ICCs ≥ 0.75 and CVs ≤ 15%, with these including cluster tendency, contrast, difference average, difference entropy, dissimilarity, homogeneity, inverse difference moment, inverse difference moment normalized, inverse difference normalized, sum entropy, and variance (Table 1).

#### GLRLM-derived RFs

The ICC assessments of 12 GLRLM-derived RFs demonstrated the variations in conditions to have substantial influence on the reliability of individual RF values (Fig. 5). With variable quantization range, no RF showed an ICC ≥ 0.75. When the bin number was varied, 10 (83%) RFs demonstrated ICCs ≥ 0.75. With either variable SNR or outliers, only two (17%) RFs showed ICCs ≥ 0.75. In the optimal condition of high SNR and no outliers, eight (67%) RFs had ICCs ≥ 0.75, irrespective of the quantization range and bin number options.

The CVs for the inter-lesion ratios of GLRLM-derived RFs were also strongly affected by the variations in conditions (Fig. 6). There were three (25%) RFs with CVs ≤ 15% with variable quantization range, five (42%) with CVs ≤ 15% with variable bin number, and one (8%) with a CV ≤ 15% with variable SNR. With variable outlier options, no GLRLM-derived RF showed a CV ≤ 15%. In the optimum condition of high SNR and no outliers, eight (67%) RFs showed CVs ≤ 15%, regardless of the quantization range and bin number options.

With inconsistent SNR and outlier conditions, no GLRLM-derived RF showed reliable measurement of either individual values or inter-lesion ratios (i.e., ICC ≥ 0.75 or CV ≤ 15%). By contrast, with high SNR and no outliers, five (42%) GLRLM-derived RFs showed reliable measurement according to both parameters, with these being high grey-level run emphasis, total number of runs, run percentage, short run emphasis and short run high grey-level emphasis (Table 2).

**Table 2:** Reliability of GLRLM-derived RFs in conditions with high SNR and no outliers.

Radiomic features	ICC		CV (%)	
	Quantization range	Bin number	Quantization range	Bin number
Grey-Level Nonuniformity	0.47–0.95	0.88–0.92	0–17	4–28
High Grey-Level Run Emphasis^*^	0.88–0.89	0.99–0.99	6–14	0–0
Long Run Emphasis	0.56–0.99	0.97–0.99	0–2	0–5
Long Run Low Grey-Level Emphasis	0.72–0.93	0.99–0.99	6–17	0–6
Long Run High Grey-Level Emphasis	0.92–0.94	0.98–0.99	21–90	8–69
Low Grey-Level Run Emphasis	0.95–0.97	0.99–0.99	3–63	2–59
Total number of runs^*^	0.89–0.98	0.81–0.86	0–0	0–1
Run Length Nonuniformity	0.64–0.94	0.82–0.87	0–1	0–5
Run Percentage^*^	0.89–0.98	0.93–0.93	0–0	0–1
Short Run Emphasis^*^	0.76–0.99	0.98–0.99	0–0	0–1
Short Run High Grey-Level Emphasis^*^	0.87–0.90	0.99–0.99	6–14	0–1
Short Run Low Grey-Level Emphasis	0.96–0.97	0.99–0.99	4–56	3–55

Note.—*RFs with ICCs ≥ 0.75 and CVs of ≤15%.

## Discussion

We experimentally investigated alterations in the histogram and probability matrices of second-order RFs in relation to variations in quantization range, bin number, noise level (i.e., SNR), and inclusion of outliers. We then subsequently performed the reliability tests with variations in these parameters in tumours with the different proportions of necrosis, evaluating the reliability and consistency of RF values and inter-lesion RF ratios. We found that all tested parameters resulted in the unreliable RFs, with inclusion of outliers particularly altering the histogram parameters (i.e., mean or SD) and distribution of the probability matrix, and consequently demonstrating unreliable measurement of RFs and inconsistent comparisons of inter-tumoural heterogeneity. Variations in SNR affected the variability of RFs in our study, as widely shown in phantom-based studies^13,23^. Considering that SNR and outliers demonstrated strong effects on the RF reliability and quantification of inter-tumoural heterogeneity, achieving appropriate SNR levels on image acquisition and excluding unintended outliers from the segmentation must take precedence for improving the reliability of RF measurements.

The impact of noise on radiomics measurements has been extensively evaluated, in relation to differing tube current, slice thickness resampling, reconstruction kernel, and reconstruction algorithms^13,24–28^. With regard to the influence of SNR on second-order features, our simulation demonstrated that the addition of noise dispersed the probability distribution, in keeping with previous literature^13^. We interpret this effect to be a result of the noise-induced increase in the SD of the image data. Interestingly, the degree of dispersion was greater in the quantization range of mean ± 2 SD than in the other quantization ranges. It means that a narrow quantization range is more vulnerable to variations in image noise. Therefore, the quantization range should be carefully applied with consideration of its relationship with the SNR level. Furthermore, efforts should be made to reduce variations in image acquisition and processing, to achieve generalizable and stable results.

Outlier control is an important factor influencing RFs^29^, although to our knowledge, there have been only limited studies evaluating how outliers affect the reliability of RFs^16,30^. Our simulation results demonstrated that the unintended inclusion of outliers within ROIs seems to have the strongest impact on the GLCM and GLRLM. The addition of an outlier strongly concentrated the probability distribution in all quantization ranges. Moreover, in the matrices with the min‒max quantization range, the distribution was shifted to the upper left direction in the GLCM, and to the left in the GLRLM. As expected, this left-sided shift was not observed with the quantization range of mean ± 2 SD or mean ± 3 SD, because the effect of the outliers truncated with these settings. Although the quantization range of mean ± 3 SD was used for outlier removal^30^ and provided more reliable information, the effects of outliers could not be fully removed by the normalization process, as demonstrated in the current study. Therefore, to achieve reliable RFs, outlier control should be carefully performed as part of the image segmentation process. Moreover, the presence of outliers should be identified with reference to histograms and probability matrices.

Contrary to SNR or the inclusion of outliers, the quantization range and bin number are conditions chosen by observers in the process of feature extraction. In a PET-based study, Hatt *et al*.^31^ suggested that resampling over 64 bins did not provide additional information. The Image Biomarker Standardization Initiative mentioned that GLCM features may be better modelled with a higher number of grey levels (i.e., 32 or 64), whereas grey level size zone matrix features would be better characterized by a lower number of grey levels (8 or 16)^32^. Similarly, Li *et al*. mentioned that robust GLCM or GLRLM features could be achieved using their own parameter settings rather than at fixed parameters in regards to quantization range and bin number^15^. It is important to understand the variability caused by these parameters and to identify the optimal image processing parameters for a specific purpose and imaging modality. In addition, the quantization range and bin number may determine the impact of low SNR and outlier inclusion, as shown in the current study. Therefore, the quantization range and bin number should be carefully chosen on the basis of an understanding of the raw data profile and the purpose of the RF extraction.

Although a number of studies have addressed issues regarding the reliability of RFs under varying parameters of image acquisition, processing, segmentation, and feature extraction^13,15–17,24,25,27,33^, there have been few studies discussing the interactions between quantization range, bin number, SNR, and outlier inclusion, as performed in our study. We evaluated histograms and probability matrices under varying parameters, demonstrating their potential interactions, to improve the understanding of the fundamental mechanisms of variability. In addition, we simulated the impact of unintended outliers and demonstrated the importance of outlier removal on image segmentation, as well as image processing. Furthermore, our experimental study was based on the image data of patients with the different necrotic features and evaluated the consistency of RFs representing inter-tumoural heterogeneity. This experimental design may more sensitively reflect changes in the heterogeneity of human tissues than phantom-based studies.

The current study has a few limitations. First, the number of cases used in our study was low. As our study aimed to demonstrate the consistency of RFs representing inter-tumour heterogeneity on the basis of imaging features extracted under 81 combinations of four conditions as a pre-clinical design, we classified the imaging features of patients into three groups and used the representative image data. Additional clinical studies are required to show the associations between clinical outcomes and tumour heterogeneity measurements under optimal settings for RF calculation. In addition, this study was based on CT data only, and the features suggested as reliable in our analysis may not be consistent across other modalities. Another limiting factor is that we generated the outlier-containing image matrix by replacing clusters of voxels in the original images, and therefore we will have lost some information from the patient data, which may act as a confounding factor. Furthermore, many other parameters such as the intra- or inter-observer reliability of image segmentation or the use of filters or kernels, were not evaluated in this study, and their effects on the reliability of RFs should be investigated in upcoming studies.

In conclusion, this study demonstrated that the reliability of RFs for evaluating tumour heterogeneity depends highly on the image quality and image processing conditions, including the quantization range, bin number, SNR, and presence of outliers, and illustrates the fundamental mechanisms of alteration to histograms and second-order RF matrices under varying parameters. In particular, the inclusion of outliers within ROIs showed a significant impact on the reliability of RFs and their inter-lesion ratios, hampering the robust evaluation of tumour heterogeneity. Optimal image SNR and the removal of outliers are crucial requirements for achieving robust and reproducible radiomics measurements, while relevant options for quantization range and bin number should be applied according to study purpose. The usefulness of RF assessment in medical imaging studies would be improved by making efforts to ensure that these factors are optimized.

## Methods

### Lesion selection and image matrix generation

This study was approved by the institutional review board of Asan Medical Center and the requirement for informed consent was waived. The imaging data were de-identified in accordance with the Health Insurance Portability and Accountability Act (HIPAA) privacy rule. All methods were performed in accordance with relevant guidelines and regulations. Eighty-three patients who underwent immunotherapy for metastatic urothelial carcinoma between October 2015 and November 2017 were collected from the electronic database of our institution. In these 83 patients, 144 lymph nodes demonstrating various proportions of necrosis were identified by a board-certified experienced radiologist (K.J.P.). The extent of necrosis was visually assessed as a semantic feature defined as a lexicon to describe regions of interest (ROI) according to the radiologist’s “eye”^1^. From these estimates of necrosis extent, three representative lymph node types with different proportions of necrosis were identified, based on the following knowledge that such features are of prognostic value^1^: (a) tumour necrosis is closely related to the biological aggressiveness and patient prognosis^34^; (b) tumour necrosis manifests as hypoattenuation on contrast-enhanced CT relative to non-necrotic tissue; and (c) heterogeneous CT values within metastatic lymph nodes are predominantly induced by different levels of intra-tumoural necrosis. The CT features of these three lesion types were as follows: (a) a mass with no or mild intratumoural heterogeneity demonstrating homogeneous enhancement; (b) a mass with intermediate heterogeneity, demonstrating multifocal hypoattenuations in 40% of the tumour area; and (c) a mass with severe heterogeneity demonstrating hypoattenuations in 70% or more of the tumour area (Fig. 1).

The radiologist drew ROIs encompassing the lesions while attempting to avoid including air, fat, vessel, and bone. Three image matrices with varying degrees of tumour heterogeneity were then generated from the ROIs (Fig. 1).

### Image acquisition

Contrast-enhanced CT images were obtained using 64-channel multidetector CT scanners (Somatom Definition AS; Siemens Medical Systems) with the following parameters: voltage, 120 kV; effective tube current, 200 mAs; scan delay, 120 sec; voxel size, 0.6 mm^3^, slice thickness, 5 mm; pitch, 1; and gantry rotation speed, 0.5 s.

### RF calculation

The RFs were calculated using Matlab software (version R2018b, The Mathworks Inc., Natick, MA, USA), and included 17 first-order and 38 second-order RFs^32^ (Supplementary Table 3). The second-order RFs were extracted from GLCM (n = 26)^35^ and GLRLM (n = 12)^36^. To test the reliability of the RFs under varying conditions of quantization range, bin number, SNR level, and the presence or absence of outliers, three options were applied for each condition; three quantization ranges (min‒max, mean ± 2 SD and mean ± 3 SD), three bin numbers (32, 64, and 128), three SNR levels (high, intermediate, and low SNR), and three outlier options (no outliers, inclusion of outliers equivalent to air or bone). In total, each RF had 81 variations (three quantization ranges × three bin numbers × three levels of SNR × three outlier options = 3^4^) according to combinations of the above calculation conditions.

### Details of RF calculation conditions

#### Quantization range and bin number

It was reported that the grey-level normalization method used affected the selected features and their classification performance^16^. The three different quantization ranges evaluated were min‒max, mean ± 2 SD and mean ± 3 SD, where min‒max represented the full range from the minimum to maximum values of the grey levels inside the ROIs (all grey levels were included), and the mean ± 2 SD and mean ± 3 SD options excluded grey levels outside the range of mean ± 2 (or 3) SD.

The fixed bin number method in which grey level intensities are discretized to a fixed number of bins allows for direct comparisons of RFs across multiple ROIs^30,32^. Based on our literature review^10,20^, the three widely used bin numbers of 32, 64 and 128 were assigned.

#### SNR

To assess the effect of SNR on the reliability of RFs, three different levels of SNR were generated by adding white noise to the original CT images. On the basis of the SD of the original CT images being an average of 5.5 HU on the three CT images, white noise was added to increase the SD to 6.5 and 8.5 HU, which reduced the SNR to 92% (intermediate SNR) and 65% (low SNR), respectively, of that of the original image (high SNR). Consequently, RF features were then calculated from these images with different SNR level.

#### Outlier

During segmentation, unintended inclusion of adjacent structures other than tumor such as bone or lung can affect the reliability of RFs. Such outliers may be accidentally included in a very low number of voxels at the periphery of ROIs during segmentation. To analyse the effect of outliers unintentionally included in ROIs, the original CT values located in a 2 × 2 voxel cluster located in the upper-left boundary of the image matrices were replaced by values equivalent to air (−950 HU) or bone (388 HU) (Fig. 1). Consequently, RFs were calculated from three outlier conditions (no outliers, air-equivalent outliers, and bone-equivalent outliers).

### Evaluation of the simulation experiments

To demonstrate the effects of changes to the imaging and segmentation quality, the interactions between the imaging parameters, and alterations to the histograms and the probability matrices were simulated for a CT image of scattered intratumoural necrosis, because they are the fundamental components for the calculation of the first- and second-order RFs (i.e., GLCM and GLRLM). As the quantization range and bin number are factors affecting the second-order RFs, their effects were only simulated on the probability matrix. Consequently, four histograms were constructed as follows: (a) high SNR without outliers, (b) low SNR without outliers; (c) high SNR with outliers equivalent to bone; and (d) low SNR with outliers equivalent to bone. In addition, the mean, median, standard deviation, and ranges of grey levels were calculated to understand the mechanisms of the RF fluctuations.

To demonstrate alterations in the probability matrix according to imaging and segmentation conditions, the following conditions were simulated: two setting (a) with the bin number fixed at 64, three quantization ranges (min‒max, mean ± 2 SD, and mean ± 3 SD), two levels of SNR (high and low), and two outlier options (no outliers and bone-equivalent outliers) were applied; and (b) with fixed settings of quantization range of the mean ± 3 SD, high SNR and no outliers, three different bin numbers (32, 64, and 128) were applied. Therefore, a total of 30 combinations of probability matrix were constructed to demonstrate changes according to differing imaging, segmentation, and processing options.

### Reliability testing

#### Reliability statistics

To test the reliability of RFs for assessing tumour heterogeneity, reliability tests were performed to evaluate the stability of each RF values and consistency of the inter-lesion RF ratios for the three image matrices with different proportions of tumour necrosis and the various simulated image quality and processing parameters. Here, inter-lesion RF ratios were defined as the ratios of each RF value across the lesions showing different proportions of tumour necrosis (i.e., ratio of lesion with no or mild heterogeneity to lesion with intermediate heterogeneity; ratio of lesion with intermediate heterogeneity to lesion with severe heterogeneity; and ratio of lesion with no or mild heterogeneity to lesion with severe heterogeneity). We hypothesized that optimal radiomics features should show consistency in these inter-lesion ratios.

First, the ICCs of RF values were calculated with one image processing option being varied and the others fixed, as previously described^15^. For example, the impact of quantization range was analyzed by varying the quantization range with the other conditions of bin number, SNR and outlier options fixed. For the ICC measurements, two-way random models (i.e., ICC [C,1)) were applied as follows^37^:

$$\frac{M{S}_{R}-M{S}_{E}}{M{S}_{R}+(k-1)M{R}_{E}},$$

where MS_R_ = mean square for rows, MS_E_ = mean square error, and k = number of observations. The reliability for measuring each RF value was assessed using the ICCs, with ICCs equal to or greater than 0.75 being taken to indicate acceptable reliability.

In addition, the reliability of RFs was assessed in terms of inter-lesion RF ratios between tumors with different proportions of heterogeneity. The consistency of inter-lesion RF ratios was evaluated using coefficients of variation (CVs), with the CVs equal to or less than 15% being taken to indicate acceptable reliability. The CV was defined as the ratio of the SD to the mean.

## Supplementary information

Supplementary Tables

## Data Availability

All software code to run the experiments used to produce all the results presented in this work is freely shared on the GitHub website at: https://github.com/HEOCHANGHOE/Asan_RF.
