# Supplementary material for: Reliability of CT radiomic features reflecting tumour heterogeneity according to image quality and image processing parameters
Source: Sci Rep. 2020 Mar 2;10:3852. doi: 10.1038/s41598-020-60868-9 (PMC7052198; doi:10.1038/s41598-020-60868-9)
Supplement: Supplementary file 1 — Supplementary Tables [file 41598_2020_60868_MOESM1_ESM.docx]

**Supplementary Materials**

**Reliability of CT radiomic features reflecting tumour heterogeneity according to image quality and image processing parameters**

Bum Woo Park^1†^, Jeong Kon Kim^1,2†^, Changhoe Heo^1^, Kye Jin Park ^2^

^1^ Asan Institute for Life Sciences, University of Ulsan College of Medicine, Seoul, 05505, South Korea

^2^ Department of Radiology and Research Institute of Radiology, University of Ulsan College of Medicine, Asan Medical Center, Seoul, Korea

† Bum Woo Park and Jeong Kon Kim are two first authors, because they equally contributed to all parts of this study

Corresponding author: Kye Jin Park, M.D.

Correspondence to [kyejin629@gmail.com](mailto:kyejin629@gmail.com)

**Supplementary Table 1. Reliability of first-order RFs in varying SNR and outliers**

| **Radiomic features** | **ICC** | |
| --- | --- | --- |
|  | **Varying SNR** | **Varying outliers** |
| Range cover | 0.36–1.00 | 0.69–1.00 |
| Energy | 0.69–1.00 | 0.71–1.00 |
| Entropy | 0.38–1.00 | 0.29–1.00 |
| Kurtosis | 0.44–1.00 | 0.09–1.00 |
| Max | 0.42–1.00 | 0.21–1.00 |
| Mean | 0.69–1.00 | 0.70–1.00 |
| Mean/median absolute deviation†‡ | 0.85–1.00 | 0.82–1.00 |
| Median | 0.74–1.00 | 0.74–1.00 |
| Min | 0.33–1.00 | 0.46–1.00 |
| Counts†‡ | 0.91–1.00 | 0.91–1.00 |
| Range | 0.71–1.00 | 0.50–1.00 |
| Root mean squared value | 0.73–1.00 | 0.72–1.00 |
| Skewness† | 1.00–1.00 | 0.52–1.00 |
| Standard deviation† | 0.84–1.00 | 0.49–1.00 |
| Sum†‡ | 0.98–1.00 | 0.98–1.00 |
| Uniformity | 0.52–1.00 | 0.26–1.00 |
| Variance | 0.63–1.00 | 0.43–1.00 |

†RFs with ICCs of 0.75 or higher in conditions of varying SNR

‡RFs with ICCs of 0.75 or higher in conditions of varying outliers

**Supplementary Table 2. Reliability of first-order RFs in conditions with high SNR and no outliers**

| **Radiomic features** | **ICC** | |  | **CV (%)** | |
| --- | --- | --- | --- | --- | --- |
|  | **Quantization range** | **Bin number** |  | **Quantization range** | **Bin number** |
| Range cover^*^ | 1.00–1.00 | 1.00–1.00 |  | 0–0 | 0–0 |
| Energy^*^ | 1.00–1.00 | 1.00–1.00 |  | 0–0 | 0–0 |
| Entropy^*^ | 0.75–1.00 | 1.00–1.00 |  | 0–0 | 1–4 |
| Kurtosis^*^ | 1.00–1.00 | 1.00–1.00 |  | 0–0 | 0–0 |
| Max^*^ | 1.00–1.00 | 1.00–1.00 |  | 0–0 | 0–0 |
| Mean^*^ | 1.00–1.00 | 1.00–1.00 |  | 0–0 | 0–0 |
| Mean/median absolute deviation^*^ | 1.00–1.00 | 1.00–1.00 |  | 0–0 | 0–0 |
| Median^*^ | 1.00–1.00 | 1.00–1.00 |  | 0–0 | 0–0 |
| Min^*^ | 1.00–1.00 | 1.00–1.00 |  | 0–0 | 0–0 |
| Counts^*^ | 1.00–1.00 | 1.00–1.00 |  | 0–0 | 0–0 |
| Range^*^ | 1.00–1.00 | 1.00–1.00 |  | 0–0 | 0–0 |
| Root mean squared value^*^ | 1.00–1.00 | 1.00–1.00 |  | 0–0 | 0–0 |
| Skewness^*^ | 1.00–1.00 | 1.00–1.00 |  | 0–0 | 0–0 |
| Standard deviation^*^ | 1.00–1.00 | 1.00–1.00 |  | 0–0 | 0–0 |
| Sum^*^ | 1.00–1.00 | 1.00–1.00 |  | 0–0 | 0–0 |
| Uniformity^*^ | 1.00–1.00 | 1.00–1.00 |  | 0–0 | 5–13 |
| Variance^*^ | 1.00–1.00 | 1.00–1.00 |  | 0–0 | 0–0 |

Note:―*RFs with ICCs of 0.75 or higher and CVs of 15% or less.

**Supplementary Table 3. First- and Second-order RFs and their equations**

|  | Name | Equation |
| --- | --- | --- |
| First-order histogram features | Range cover | $Maximum:=max(X)$ |
|  | Energy | $Energy := \sum_{i}^{N_{l}} \boldsymbol{X}\left( i \right)^{2}$ |
|  | Entropy | $Entropy :=- \sum_{i}^{N_{l}} \boldsymbol{P}\left( i \right)*\log_{2} \boldsymbol{P}\left( i \right)$ |
|  | Kurtosis | $Kurt := \frac{\sum_{i}^{N_{l}} \boldsymbol{P}\left( i \right)*\left( \boldsymbol{B}\left( i \right)- \overline{X} \right)^{4}}{\left( \sqrt{\sum_{i}^{N_{l}} \boldsymbol{P}\left( i \right)*\left( \boldsymbol{B}\left( i \right)-\overline{X} \right)} \right)^{4}}$ |
|  | Max | $Maximum:=max(X)$ |
|  | Mean | $Mean = \overline{X}:= \frac{1}{N}\sum_{i}^{N} X(i)$ |
|  | Mean/median absolute deviation | $MAD := \frac{1}{N}\sum_{i}^{N} \vert X\left( i \right)-\bar{X\vert}$ |
|  | Median | The median gray level intensity within the$X(i)$ |
|  | Min | $Minimum:= min(X)$ |
|  | Counts | Pixel counts within the$X(i)$ |
|  | Range | $Range:=\max\left( X \right)-min(X)$ |
|  | Root mean squared value | $RMS:=\sqrt{\frac{1}{N}\sum_{i}^{N} (X\left( i \right)+c)^{2}}$ |
|  | Skewness | $Skew := \frac{\sum_{i}^{N_{l}} \boldsymbol{P}\left( i \right)*\left( \boldsymbol{B}\left( i \right)- \overline{X} \right)^{3}}{\left( \sqrt{\sum_{i}^{N_{l}} \boldsymbol{P}\left( i \right)*\left( \boldsymbol{B}\left( i \right)-\overline{X} \right)} \right)^{3}}$ |
|  | Standard deviation | $StdDev := \sqrt{\frac{1}{N-1}\sum_{i}^{N} \left( \boldsymbol{X}\left( i \right)- \overline{X} \right)^{2}}$ |
|  | Sum | $Sum := \sum_{i}^{N_{l}} \boldsymbol{X}(i)$ |
|  | Uniformity | $Uniformity := \sum_{i}^{N_{l}} \boldsymbol{P}\left( i \right)^{2}$ |
|  | Variance | $CV := \frac{standard deviation}{mean}$ |
| GLCM features | Angular second moment | $ASM:=\sum_{i}^{N_{g}} \sum_{j}^{N_{g}} \boldsymbol{P}\left( i,j \right)^{2}$ |
|  | Autocorrelation | $ACO :=\sum_{i}^{N_{g}} \sum_{j}^{N_{g}} i*j*\boldsymbol{P}(i,j)$ |
|  | Cluster Prominence | $CLP :=\sum_{i}^{N_{g}} \sum_{j}^{N_{g}} \left( i+j-u_{x}-u_{y} \right)^{4}\boldsymbol{*P}\left( i,j \right)$ |
|  | Cluster Shade | $CLS :=\sum_{i}^{N_{g}} \sum_{j}^{N_{g}} \left( i+j-u_{x}-u_{y} \right)^{3}\boldsymbol{*P}\left( i,j \right)$ |
|  | Cluster Tendency | $CLT :=\sum_{i}^{N_{g}} \sum_{j}^{N_{g}} \left( i+j-u_{x}-u_{y} \right)^{2}\boldsymbol{*P}\left( i,j \right)$ |
|  | Contrast | $CON :=\sum_{i}^{N_{g}} \sum_{j}^{N_{g}} \left( i-j \right)^{2}\boldsymbol{*P}(i,j)$ |
|  | Correlation | $CORR :=\sum_{i}^{N_{g}} \sum_{j}^{N_{g}} \frac{\left( i-u_{x} \right)\boldsymbol{*}\left( j-u_{y} \right)}{\boldsymbol{\sigma}_{\boldsymbol{x}}\boldsymbol{\sigma}_{\boldsymbol{y}}}\boldsymbol{*P}\left( i,j \right)$ |
|  | Difference Average | $DIFA :=\sum_{i}^{N_{g}} {i*\boldsymbol{P}}_{\boldsymbol{x-y}}(i)$ |
|  | Difference entropy | $DIFE :=\sum_{i}^{N_{g}} \boldsymbol{P}_{\boldsymbol{x-y}}\left( i \right)*\log_{2} (\boldsymbol{P}_{\boldsymbol{x-y}}\left( i \right))$ |
|  | Difference variance | $DIFV :=\sum_{i}^{N_{g}} \left( i- DIFA \right)^{2}*\boldsymbol{P}_{\boldsymbol{x-y}}(i)$ |
|  | Dissimilarity | $Diss := \sum_{i}^{N_{g}} \sum_{j}^{N_{g}} \boldsymbol{\vert i-j\vert*P}\left( i,j \right)$ |
|  | Entropy | $ENT :=-\sum_{i}^{N_{g}} \sum_{j}^{N_{g}} \boldsymbol{P}\left( i,j \right){*log}_{2} \left[ \boldsymbol{P}\left( i,j \right) \right]$ |
|  | Harralick Correlation | $Harral :=\sum_{i}^{N_{g}} \sum_{j}^{N_{g}} \frac{\boldsymbol{i*j*P}\left( i,j \right)-(u_{x}\boldsymbol{*}u_{y})}{\boldsymbol{\sigma}_{\boldsymbol{x}}\boldsymbol{\sigma}_{\boldsymbol{y}}}$ |
|  | Homogeneity | $HOM :=\sum_{i}^{N_{g}} \sum_{j}^{N_{g}} \frac{\boldsymbol{P}(i,j)}{1+ \left\vert i-j \right\vert}$ |
|  | Informational measure of correlation 1 | $IMC1 :=\frac{HXY-HXY1}{\max\left( HX, HY \right)}$  $, where HX :=-\sum_{i}^{N_{g}} P_{x}\left( i \right)*\log\left[ P_{y}\left( i \right) \right]$  $HY :=-\sum_{j}^{N_{g}} P_{y}\left( j \right)*\log\left[ P_{y}\left( j \right) \right]$  $HXY :=-\sum_{i}^{N_{g}} \sum_{j}^{N_{g}} \boldsymbol{P}\left( i,j \right)*log \left[ \boldsymbol{P}\left( i,j \right) \right]$  $HXY1 :=-\sum_{i}^{N_{g}} \sum_{j}^{N_{g}} \boldsymbol{P}\left( i,j \right)*\log\left[ P_{x}\left( i \right)\boldsymbol{*}P_{y}\left( j \right) \right]$  $HXY2 :=-\sum_{i}^{N_{g}} \sum_{j}^{N_{g}} P_{x}\left( i \right)\boldsymbol{*}P_{y}\left( j \right)*\log\left[ P_{x}\left( i \right)\boldsymbol{*}P_{y}\left( j \right) \right]$ |
|  | Informational measure of correlation 2 | $IMC2 :=\sqrt{1-e^{-2(HXY2-HXY)}}$ |
|  | Inverse difference moment | $IDM :=\sum_{i}^{N_{g}} \sum_{j}^{N_{g}} \frac{\boldsymbol{P}(i,j)}{1+\left( i-j \right)^{2}}$ |
|  | Inverse difference moment normalized | $IDMN :=\sum_{i}^{N_{g}} \sum_{j}^{N_{g}} \frac{\boldsymbol{P}(i,j)}{1+\left( i-j \right)^{2}/N^{2}}$ |
|  | Inverse difference normalized | $IDN :=\sum_{i}^{N_{g}} \sum_{j}^{N_{g}} \frac{\boldsymbol{P}(i,j)}{1+ \left\vert i-j \right\vert/N}$ |
|  | Inverse Variance | $IVAR :=\sum_{i}^{N_{g}} \sum_{j}^{N_{g}} \frac{\boldsymbol{P}(i,j)}{\left( i-j \right)^{2}}\boldsymbol{;}i \neq j$ |
|  | Maximum Probability | $MAXP :=\max\{\boldsymbol{P}\left( i,j \right)\}$ |
|  | Mean | $MEAN :=\sum_{i}^{N_{g}} \sum_{j}^{N_{g}} i*\boldsymbol{P}(i,j)$ |
|  | Sum average | $SUMA:=\sum_{i}^{{2N}_{g}} {i*\boldsymbol{P}}_{\boldsymbol{x+y}}\left( i \right)$ |
|  | Sum entropy | $SUME :=-\sum_{i}^{{2N}_{g}} \boldsymbol{P}_{\boldsymbol{x+y}}\left( i \right)*\log_{2} (\boldsymbol{P}_{\boldsymbol{x+y}}\left( i \right))$ |
|  | Sum variance | $SUMV :=\sum_{i}^{{2N}_{g}} \left( i- SUMA \right)^{2}*\boldsymbol{P}_{\boldsymbol{x+y}}(i)$ |
|  | Variance | $VAR :=\sum_{i}^{N_{g}} \sum_{j}^{N_{g}} \left( i-MEAN \right)^{2}\boldsymbol{*P}(i,j)$ |
| GLRLM features | Gray-Level Non-uniformity | $GLN:= \frac{1}{N_{run}}\sum_{k}^{N_{g}} \left[ \sum_{l}^{N_{r}} \boldsymbol{P}\left( k,l \right) \right]^{2}$ |
|  | High gray-level run emphasis | $HGRE:= \frac{1}{N_{run}}\sum_{k}^{N_{g}} \sum_{l}^{N_{r}} k^{2}\boldsymbol{*P}(k,l)$ |
|  | Long run emphasis | $LRE := \frac{1}{N_{run}}\sum_{k}^{N_{g}} \sum_{l}^{N_{r}} l^{2}*\boldsymbol{P}(k,l)$ |
|  | Long Run Low Gray-Level Emphasis | $LRLGE := \frac{1}{N_{run}}\sum_{k}^{N_{g}} \sum_{l}^{N_{r}} \frac{l^{2}}{k^{2}}\boldsymbol{P}(k,l)$ |
|  | Long Run High Gray-Level Emphasis | $LRHGE := \frac{1}{N_{run}}\sum_{k}^{N_{g}} \sum_{l}^{N_{r}} k^{2}l^{2}*\boldsymbol{P}(k,l)$ |
|  | Low gray-level run emphasis | $LGRE:= \frac{1}{N_{run}}\sum_{k}^{N_{g}} \sum_{l}^{N_{r}} \frac{1}{k^{2}}\boldsymbol{P}(k,l)$ |
|  | Total number of runs | $TNR := \sum_{k}^{N_{g}} \sum_{l}^{N_{r}} \boldsymbol{P}(k,l)$ |
|  | Run Length Non-uniformity | $RLN := \frac{1}{N_{run}}\sum_{l}^{N_{r}} \left[ \sum_{k}^{N_{g}} \boldsymbol{P}\left( k,l \right) \right]^{2}$ |
|  | Run Percentage | $RP:= \frac{N_{run}}{Number of voxels in ROI}$ |
|  | Short run emphasis | $SRE:=\frac{1}{N_{run}}\sum_{k}^{N_{g}} \sum_{l}^{N_{r}} \frac{1}{l^{2}}*\boldsymbol{P}(k,l)$ |
|  | Short Run High Gray-Level Emphasis | $SRHGE:= \frac{1}{N_{run}}\sum_{k}^{N_{g}} \sum_{l}^{N_{r}} \frac{k^{2}}{l^{2}}\boldsymbol{P}(k,l)$ |
|  | Short Run Low Gray-Level Emphasis | $SRLGE:= \frac{1}{N_{run}}\sum_{k}^{N_{g}} \sum_{l}^{N_{r}} \frac{1}{{k^{2}l}^{2}}\boldsymbol{P}(k,l)$ |
